# Increased decomposer diversity accelerates and potentially stabilises litter decomposition

**DOI:** 10.1016/j.soilbio.2015.01.026

**Published:** 2015-04

**Authors:** Florian Kitz, Michael Steinwandter, Michael Traugott, Julia Seeber

**Affiliations:** Institute of Ecology, University of Innsbruck, Technikerstrasse 25, 6020 Innsbruck, Austria

**Keywords:** Sciaridae, Mesocosm, Alpine, *Lumbricus rubellus*, *Cylindroiulus fulviceps*, Biodiversity

## Abstract

Little is known about the effect of decomposer diversity on litter decomposition in alpine areas. Especially under the premise that alpine ecosystems are very sensitive to global change and are currently undergoing extensive land-use changes, a better understanding is needed to predict how environmental change will affect litter decomposition. A mesocosm experiment was conducted to compare the effects of the most common and functionally diverse invertebrates (earthworms, millipedes and sciarid larvae) found in alpine soils on decomposition rates and to assess how decomposer diversity affects litter decomposition. Experimental and estimated (i.e. projected to field decomposer-biomass) litter mass loss was 13–33% higher in the three-species treatment. Notably, the variability in decomposition was greatly reduced when decomposer diversity was high, indicating a portfolio effect. Our results suggest that invertebrate decomposer diversity is essential for sustaining litter decomposition in alpine areas and for the stability of this service.

## Highlights

• We investigated the effect of decomposer diversity on litter decomposition in alpine areas.
• We confirm the importance of functional diversity in litter decomposition processes.
• Increased decomposer diversity decreases the variability in litter mass loss.

Over the last decades the question of how diversity can alter ecosystem functioning has been a controversial topic in ecology (e.g. Balvanera et al., 2006), even though a consensus about the importance of diversity between functional levels has been reached in the last years (Scheu and Setälä, 2002; Cardinale et al., 2012). While studies dealing with the effects of diversity on decomposition in general offer opposing conclusions (Naeem et al., 1994; Srivastava et al., 2009; Bradford et al., 2014), the role of soil animals is the most disputed aspect due to scarce empirical data (Gessner et al., 2010; David, 2014). The lack of data especially concerns terrestrial ecosystems, among which diversity effects on decomposition in alpine areas are particularly poorly understood. Taking into account the anticipated strong impact of global changes on alpine ecosystems (IPCC, 2013), insight into those processes is urgently needed to better predict how changes in decomposer diversity affect ecosystem services such as litter decomposition, as e.g. climate may moderate the influence of soil fauna on decomposition (Garcia-Palacios et al., 2013). Alpine ecosystems are characterised by shallow soils, a short growth season, low temperatures and a low vegetation cover (Spehn et al., 2006). The most abundant litter decomposers present in the Central Alps are earthworms, millipedes and dipteran larvae, with Sciaridae predominating among the latter (Seeber et al., 2012). Since little quantitative information is available on the decomposition efforts of these invertebrates in alpine soils, this study examined their individual decomposition performance and investigated how litter decomposition is affected by decomposer diversity.

Decomposers were obtained in alpine and high-alpine areas (Kaserstattalm, Tyrol, Austria 47.12601°N 11.29092°E). Adult sciarids (mainly *Bradysia* spp.) were used to set up a culture of larvae using the experimental conditions described below. Each mesocosm (plastic cup: 378 ml) contained 50 ml sieved (4 mm) and air-dried soil (pH 5.24 ± 0.05, taken from the study site) mixed with 40 ml clay balls (diameter 4–8 mm) to prevent siltation. One gram of air-dried and chopped litter of *Dactylis glomerata*, a Poaceae wide-spread in alpine ecosystems, was provided as a food source for the decomposers and placed on top of the soil.

Mesocosms were incubated at 15 °C for a 6 day pre-experimental period in order to allow microbes to establish. Thereafter, soil animals were added as shown in Table 1 (specimens of similar biomass were used), with seven replicates per treatment. Upon experimental start each mesocosm was watered once (10 ml) and kept in a climate chamber for 4 weeks, receiving 4 ml of water every 3 days. The number of adult sciarids was counted in each mesocosm when watering them to monitor the development of the larval populations. Two weeks after experimental start, 11 sciarid larvae were added per mesocosm to replace for larvae which entered pupation. After 28 days, all soil animals were removed and the litter material was separated from the soil, dried and the mass of both animals and litter was determined to the nearest 0.001 mg. One mesocosm (D + S treatment) was excluded because the millipede died during the experiment, as well as two outliers (one S and one D + L + S treatment). Litter mass loss was calculated by subtracting remaining from original litter mass; differences between treatments were analysed by ANOVA. We statistically checked for non-additive effects in the three-species treatment by comparing observed with expected values using a t-Test. All analyses and graphics were done in R 3.1.1 (R Core Team, 2014).

**Fig. 1:**
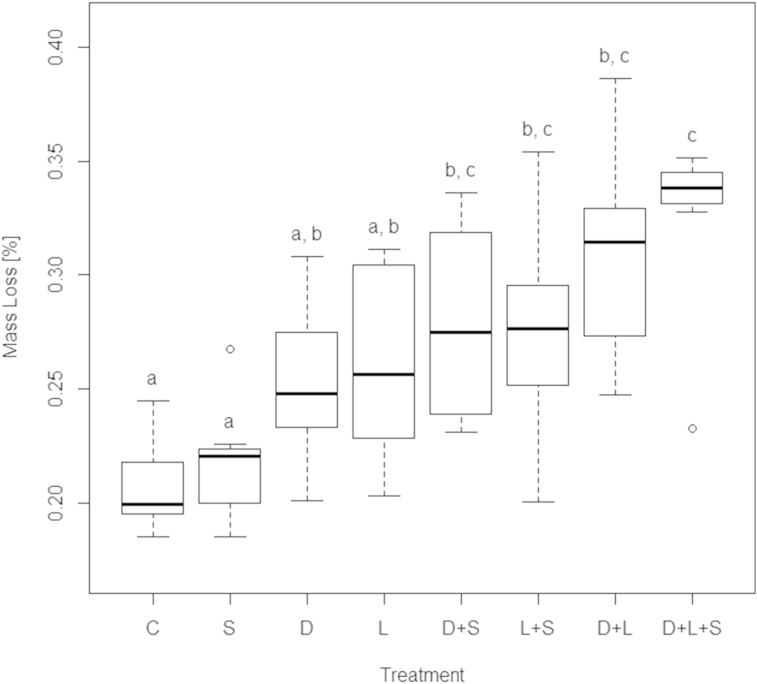
Relative mass loss of *Dactylis glomerata* litter (%) in mesocosms harbouring Diplopoda (D), Lumbricidae (L), Sciaridae (S) and respective combinations, including a control treatment without detritivores (C) after 4 weeks (n = 7).

**Fig. 2:**
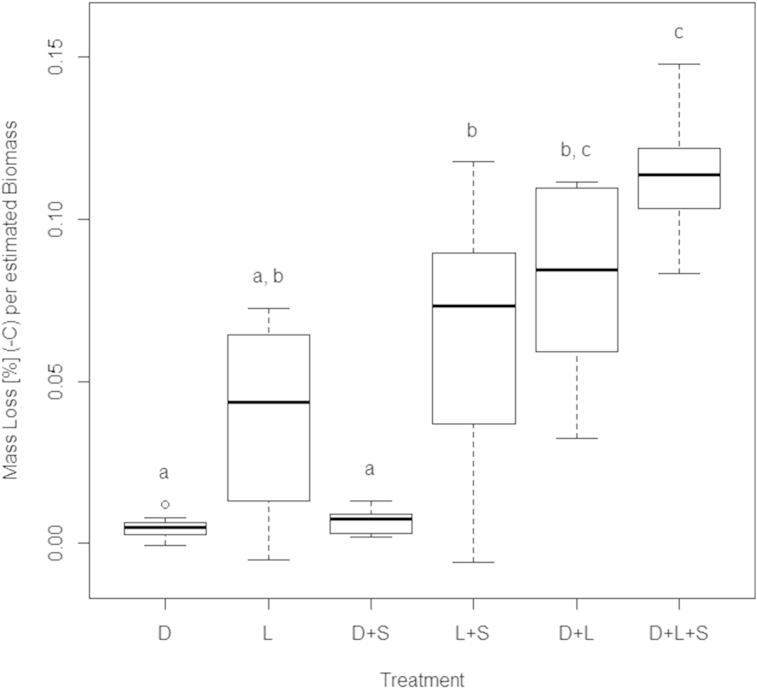
Estimated mass loss of *Dactylis glomerata* litter (%) projected to field-based decomposer densities in high alpine soils including Diplopoda(D), Lumbricidae (L), Sciaridae (S), and respective combinations after 4 weeks. Litter mass loss was calculated for each treatment by subtracting the litter mass loss of the control, then dividing it by the initial total animal biomass present in the experiment and afterwards multiplying it by the estimated biomass present in high alpine ecosystems. Different letters indicate significant differences (post-hoc test with Tukey correction following ANOVA F_5,34_ 14.95; P < 0.001).

Litter mass loss was about 20% in the control treatment, representing microbial litter decomposition, and increased in mesocosms harbouring animal decomposers by 4.6%–13.3% (Fig. 1). An exception was the Sciaridae monoculture (Dunnett Contrasts analysis P 1.00), which was therefore excluded from all further analysis. The three-species treatment showed the highest decomposition rate compared to the monotreatments, followed by the *Lumbricus rubellus* + *Cylindroiulus fulviceps* treatment (Fig. 1). Most noticeable is the low variability in the three-species treatment compared to all other treatments (however, Levene test P 0.08). To estimate litter mass loss under field conditions, experimental mass loss of each mesocosm was projected to decomposer biomass typically found in high-alpine grasslands (Seeber et al., 2012; Steinwandter, 2012) after subtracting microbial decomposition (Fig. 2). Estimated litter mass loss for communities containing earthworms was highest, while diplopod effects became less important. This can be explained by low abundances of millipedes in high-alpine soils (decomposer biomass ratio (L:D:S): experiment: 44:10:1; estimates for an alpine meadow: 1162:3:1). The litter mass loss projected for field conditions was significantly different between the three-species-treatment and the millipede monoculture (P < 0.001), the earthworm monoculture (P < 0.001), the millipede/sciarid treatment (P < 0.001) and the earthworm/Sciaridae treatment (P 0.013) (Fig. 2). Sciaridae induced a higher litter mass loss in the three-species-treatment, but showed no significant difference from the control on their own.

In accordance with recent literature which suggests that functional diversity might be more important than species richness for biodiversity-ecosystem functioning relationships (see Heemsbergen et al., 2004; Balvanera et al., 2006), we designed our experiment using three functionally diverse groups of decomposers: earthworms, millipedes and sciarid larvae. Earthworms such as *Lumbricus rubellus* fragment and mix litter with mineral soil, and their organic-mineral faeces are deposited in the litter layer. Millipedes such as *Cylindroiulus fulviceps* fragment litter and deposit their purely organic faeces in the litter layer. Due to the small size of the sciarid larvae they most probably feed on faeces of larger decomposers, thereby further advancing decomposition, a fact which fits with our observations.

The highest decomposition rates were observed in the three-species treatment, exceeding a pure additive effect of the species involved (Figs. 1 and 2, P < 0.001), thereby confirming the importance of functional diversity in litter decomposition processes (Nielsen et al., 2011). A notable result of our study is the low variability of experimental (Fig. 1) and estimated litter mass loss (Fig. 2) in the three-species treatment. This points towards a portfolio effect (Tilman, 1999), where an increased diversity of decomposers not just increases litter decomposition rates but also reduces variability and as such increases stability of this ecosystem service. This idea is corroborated by previous findings where stabilising effects of decomposer diversity on litter decomposition were demonstrated for fungal communities (Dang et al., 2005; Pascoal et al., 2010), in aquatic systems (McKie et al., 2009), and in grasslands (Eisenhauer and Schädler, 2011). Future work should examine the underlying mechanisms in greater detail to allow for better predictions on the role of invertebrate decomposer diversity for the magnitude and stability of litter decomposition in alpine areas under a changing climate and management. The current experiment did not allow us to differentiate between functional diversity and species richness per se as each of the three decomposer species had a unique functional role. Further experiments where several species within the same functional group are present will make it possible to tease apart the effects of these two components of biodiversity.

## Figures and Tables

**Table 1:** The number of individuals of the three decomposer species with mean biomass in gram and standard deviation in parentheses used in the different treatments per mesocosm. L Lumbricidae (*Lumbricus rubellus*), D Diplopoda (*Cylindroiulus fulviceps*), S sciarid larvae (*Bradysia* spp.).

Treatment	*L. rubellus*	*C. fulviceps*	Sciarid larvae
S	–	–	11 (0.013 ± 0.0011)
D	–	1 (0.11 ± 0.02)	–
L	1 (0.63 ± 0.13)	–	–
S + D	–	1 (0.15 ± 0.04)	11 (0.012 ± 0.0015)
S + L	1 (0.53 ± 0.10)	–	11 (0.010 ± 0.0009)
D + L	1 (0.46 ± 0.12)	1 (0.12 ± 0.03)	–
S + L + D	1 (0.44 ± 0.09)	1 (0.12 ± 0.04)	11 (0.012 ± 0.0010)
C	–	–	–
